# Knowledge and preventive behaviors regarding COVID-19 in Bangladesh: A nationwide distribution

**DOI:** 10.1371/journal.pone.0251151

**Published:** 2021-05-03

**Authors:** Ismail Hosen, Amir H. Pakpour, Najmuj Sakib, Nur Hussain, Firoj al Mamun, Mohammed A. Mamun

**Affiliations:** 1 CHINTA Research Bangladesh (Centre for Health Innovation, Networking, Training, Action and Research–Bangladesh), Dhaka, Bangladesh; 2 Department of Public Health and Informatics, Jahangirnagar University, Dhaka, Bangladesh; 3 Social Determinants of Health Research Center, Research Institute for Prevention of Non-Communicable Diseases, Qazvin University of Medical Sciences, Qazvin, Iran; 4 Department of Nursing, School of Health and Welfare, Jönköping University, Jönköping, Sweden; 5 Department of Microbiology, Jashore University of Science and Technology, Jashore, Bangladesh; 6 School of Earth, Environment & Society, McMaster University, Hamilton, Ontario, Canada; Iwate Medical University, JAPAN

## Abstract

Assessing individuals’ knowledge and preventive behaviors towards the Coronavirus Disease of 2019 (COVID-19) is essential for the related public health surveillance strategies. Although some of the studies were conducted in Bangladesh, none of these studies considered the geographical distribution of knowledge and preventive behaviors towards COVID-19. Therefore, the present nationwide cross-sectional study with 10,067 samples for the first-time aims to assess the knowledge gap by presenting the geographical distribution of the COVID-19 knowledge and preventive behaviors across all administrative districts of Bangladesh. The measures included socio-demographics and questions about knowledge and preventive behaviors related to COVID-19. One-way ANOVA, independent t-test, and multiple linear regression were used to analyze the data. In addition, GIS-based mapping identified district-wise distribution of the outcomes. Results indicated that the overall mean score of knowledge related to COVID-19 was 14.363 ± 3.073, whereas 16.95 ± 2.89 was for preventive behaviors. Participants’ being male, being divorced or widowed, consuming alcohol, smoking cigarettes, living in villages, and having no formal education reported lower performing preventive COVID-19 behaviors. Those participants with higher knowledge scores reported higher preventive COVID-19 behaviors (β = 0.053, *p*<0.001). However, the model predicted only 13.2% of the variation in preventive COVID-19 behaviors while the overall model being significant. The findings suggest that the Bangladeshi government should initiate appropriate far-reaching program of health education focusing on knowledge and preventive behaviors towards COVID-19 at a community level. After all, the strategies to combat COVID-19 will require individuals’ involvement to control and prevent the disease outbreak, for which education is essential.

## Introduction

The novel coronavirus that causes COVID-19 can be transmitted directly through human to human contact or indirectly with contaminated objects [1]. Symptoms include fever, dry coughing, vomiting, diarrhea, nausea, and fatigue that may result in severe problems such as difficulty in breathing, talking, moving, and chest pain [1, 2]. The virus can also be transmitted asymptomatically. The World Health Organization (WHO) included COVID-19 as an international concern to public health on 30 January 2020; the WHO later reclassified the virus as a pandemic on 11 March 2020 [3]. The first identified case of COVID-19 in Bangladesh occurred on 8 March 2020. The transmission rate was under control during the initial phase of the outbreak, although it started to increased rapidly from April [4, 5]; by early November a total of 400,251 confirmed cases were reported [6].

The government of Bangladesh adopted a rapid response by imposing about two months’ lockdown [5]. However, the lockdown could not continue; the country reported aggravated suicide incidences as of economic distress related to the lockdown [7, 8]. The authority also imposed some of the rules and restrictions related to social distancing, mandatory wearing masks, limited transport facilities and traveling, etc. as instructed by the WHO [4, 5, 9, 10]. Therefore, public participation in government strategies is mandatorily expected to minimize the country’s COVID-19 transmission rate. Moreover, the extent to which an individual adheres to the control measures is determined by their attitudes towards and knowledge of the outbreak [11, 12]. Knowledge of specific diseases acts as an essential component in public health and disease prevention and helps to convince individuals to adhere to public health measures [3]. Besides, a previous outbreak (SARS outbreak in 2003) found that community-focused health education campaigns, which fostered positive attitudes towards public health measures in individuals, helped to obstruct the risk of spreading disease [13]. Knowledge, attitudes and practices towards COVID-19, have therefore been extensively studied in several places: Clements et al. in the US [14], Li et al. in China [15], Adela et al. in Cameroon [16], Lin et al. in Iran [17], and Pagnini et al. in Italy [18], etc.

Bangladesh is a densely populated country with limited healthcare resources; the control of COVID-19 will be difficult herein without public participation. It is necessary to have data on public knowledge and preventive behaviors regarding COVID-19. This can reduce the government’s burden in combating the outbreak strategies. There are a few studies [3, 9, 19–21] that have been conducted in the country which address this issue. However, these studies are non-representative with limited sample sizes and do not provide any (i) district-wise mapping regarding knowledge and preventive behaviors, or (ii) use any models. Thus, the present study investigated the knowledge and preventive behaviors towards COVID-19 and its related factors and presented through countrywide mapping considering every administrative district throughout a nationwide survey in Bangladesh.

## Methods

A nationwide cross-sectional survey was carried out between 1 and 10 April 2020. Data were collected using online promotion instead of face-to-face interviews due to COVID-19 pandemic related restrictions. A structured questionnaire was developed and pre-tested by 250 research assistants through online media. Participation was voluntary. Prior to data collection, informed online consent was taken from the participants (or consent from the legal guardians of those younger than 18) after informing them of study’s aims. Eventually, a total of 10,067 samples were recruited for final analysis. Moreover, the detailed methodology, including ethical approvals, can be found in the previously published studies from this dataset [22–24]; it is to be noted that the articles are distinct based on the study objectives.

### Ethical statement

The ethical aspects of the present study were reviewed and approved by the Institute of Allergy and Clinical Immunology of Bangladesh ethics board, Bangladesh [IRBIACIB/CEC/03202005] and Biosafety, Biosecurity, and Ethical Committee of Jahangirnagar University, Bangladesh [BBEC, JU/M 2O20/COVlD-l9/(9)2]. Online informed consent was provided before survey participation, and the online consent form included purpose of the study, data confidentiality, rights of participation, and the right to withdraw from the study at any time. Parental or guardian consent was obtained for participants aged below 18 years.

### Measures

The survey included questions on basic socio-demographics, knowledge, and preventive behaviors related to COVID-19. The detailed questions are available in the S1 File.

### Socio-demographic factors

The basic socio-demographic information were collected such as (i) age, (ii) gender, (iii) education, (iv) occupation, (v) place of residence, (vi) marital status, (vii) smoking status, (viii) alcohol-consumption and, (ix) current health status. A single question was utilized to observe participants’ current health status using seven response choices (i.e., diabetes, cancer, heart disease, high blood pressure, kidney problem, asthma/respiratory problem, and any others not listed). The number of comorbidities was measured with a score of 1 point for each condition listed (e.g., someone with cancer, diabetes, and high blood pressure would score 3).

### Knowledge about COVID-19

A list of questions was used to measure participants’ level of knowledge about COVID-19. Knowledge was assessed based on four domains (i.e., the spread of infection, symptoms, prevention, and treatment), each consisting of true or false statements. One point was awarded for each correct answer and 0 points for an incorrect answer. Sub-totals were calculated for each domain and a total score for all the items on a range from 0–20, where higher scores indicate better knowledge. The Kuder-Richardson Formula 20 (KR-20) for 20 items of knowledge was 0.76, and the item-total correlation ranged from 0.10 to 0.50.

### COVID-19 preventive behaviours

Participants’ level of preventive behaviors related to COVID-19 was also assessed in the present survey. A total of 4-questions concerning preventive behaviors were included in the survey, where responses were recorded on a 5-point Likert scale (1 = never to 5 = almost always). A total score for all the items ranges from 4 to 20. Higher scores indicate better preventive behaviors of the participants. Internal consistency for preventive behavior was 0.721, and corrected item-total correlation ranged from 0.45 to 0.54.

### Statistical analysis

Data analyses were performed using SPSS (version 24.0). Data were presented as mean ± standard deviation for continuous data and proportions and percentages for categorical or qualitative data. To determine the association between socio-demographics, knowledge and preventive COVID-19 behaviors, one-way ANOVA and independent t-test were used. Multiple linear regression was used to analyze the factors influencing preventive COVID-19 behaviors. Socio-demographic variables were significantly associated with behavior score in univariate analyses (*p* < 0.05) and were adjusted in the regression analysis. Multicollinearity of independent variables was detected if the variance inflation factor (VIF) was more than 10. A *p*-value <0.05 was set as statistically significant. ArcGIS 10.7 was used for mapping and spatial analysis of COVID-19 knowledge and preventive behaviors. The maps were obtained from the Bangladesh Government mapping sites, where maps are provided for free use.

## Results

### Characteristics of the participants

The distribution of socio-demographic characteristics is presented in “**Table 1**”, shows that out of 10,067 participants, 56.1% were males. Most of the participants surveyed were between 20 to 29 years of age (71.3%); 58.4% were students; 40.1% lived in the divisional city; 70.3% were unmarried; and 85.2% and 97.3%, respectively, were nonsmokers and did not consume alcohol.

**Table 1:** Distribution of participant characteristics in respect to knowledge about COVID-19 and preventive COVID-19 behaviors.

Predictors	n; %	Knowledge about COVID-19			Preventive COVID-19 behaviors			
		N	Mean	SD	N		Mean	SD
**Gender**								
*Male*	5650 (56.1)	5650	14.35	3.12	5434	16.66		2.95
*Female*	4402 (43.7)	4402	14.39	2.99	4246	17.32		2.78
t-value (*p*-value)		-0.60 (0.034)			-11.12 (<0.001)			
**Age group**								
*10–19*	685 (6.8)	685	13.90	3.12	666		16.98	2.95
*20–29*	7175 (71.3)	7175	14.47	2.93	6931		17.15	2.62
*30–39*	1221 (12.1)	1221	14.61	3.14	1154		17.02	2.91
*40–49*	410 (4.1)	410	13.93	3.67	394		15.73	3.94
*50–59*	371 (3.7)	371	13.74	3.72	353		15.36	3.93
*60 and above*	196 (1.9)	196	12.96	4.12	188		14.54	4.43
f-value (*p*-value)		19.17 (<0.001)			70.66 (<0.001)			
**Educational status**								
*No formal education*	197 (2)	197	11.64	4.20	188		11.60	4.52
*Primary school*	169 (1.7)	169	12.18	3.96	154		14.05	4.66
*Secondary school*	427 (4.2)	427	13.50	3.36	405		15.89	3.62
*Higher secondary*	1139 (11.3)	1139	13.88	3.27	1090		16.50	3.15
*Tertiary education*	8135 (80.8)	8135	14.59	2.91	7858		17.25	2.53
f-value (*p*-value)		89.33 (<0.001)			266.15 (<0.001)			
**Occupational status**								
*Unemployed*	361 (3.6)	361	14.27	3.38	354		16.68	3.23
*Employed*	2586 (25.7)	2586	14.38	3.22	2459		16.79	3.06
*Retired*	92 (0.9)	92	14.33	3.75	90		16.47	3.59
*Housewife*	1150 (11.4)	1150	14.04	3.37	1096		16.42	3.52
*Student*	5878 (58.4)	5878	14.42	2.91	5696		17.14	2.64
f-value (*p*-value)		3.82 (0.004)			18.77 (<0.001)			
**Residence area**								
*Village*	2336 (23.2)	2336	13.86	3.19	2242		15.94	3.48
*Sub-district town*	1359 (13.5)	1359	14.19	3.10	1316		17.04	2.78
*District town*	2334 (23.2)	2334	14.31	3.16	2245		17.32	2.65
*Divisional city*	4038 (40.1)	4038	14.74	2.89	3892		17.29	2.55
f-value (*p*-value)		42.92 (<0.001)			125.61(<0.001)			
**Marital status**								
*Single*	7081 (70.3)	7081	14.41	2.95	6843		17.13	2.65
*Married*	2839 (28.2)	2839	14.34	3.27	2709		16.62	3.28
*Divorced/widowed*	147 (1.4)	147	12.78	4.19	143		14.35	4.53
f-value (*p*-value)		20.45(<0.001)			91.18(<0.001)			
**Currently smoker**								
*Yes*	1486 (14.8)	1486	14.42	3.37	1419		16.23	3.371
*No*	8581 (85.2)	8581	14.35	3.02	8276		17.07	2.79
t-value (*p*-value)		0.751 (<0.001)			-10.12 (<0.001)			
**Alcohol use**								
*Yes*	267 (2.7)	267	13.99	3.79	258		15.12	3.91
*No*	9800 (97.3)	9800	14.37	3.05	9437		17.00	2.85
t-value (*p*-value)		-2.02 (<0.001)			-10.35 (<0.001)			

### Knowledge about COVID-19

The overall mean score of knowledge related to COVID-19 was 14.363 ± 3.073. The COVID-19 knowledge score was significantly higher among the participants (i) being females (14.39 ± 2.99, t = -0.60, *p* = 0.03) (ii) aged between 30 to 39 years of age (14.61± 3.14, *f* = 19.17, *p*<0.001); (iii) had higher level of education (14.59 ± 2.91, *f* = 89.33, *p*<0.001); (iv) were students (14.42± 2.91, *f* = 3.82, *p* = 0.004); (v) belonged to divisional city (14.74 ± 2.89, f = 42.92, *p*<0.001), (vi) unmarried (14.41 ± 2.95, *f* = 20.45, *p*<0.001), (vii) smoker (14.42 ± 3.37, t = 0.751, *p*<0.001) and (viii) non-alcoholic individuals (14.37 ± 3.05, t = -2.02, *p*<0.001) (**Table 1**).

### Preventive behaviors against COVID-19

The overall mean score of preventive COVID-19 behaviors was 16.95 ± 2.89. The mean score of preventive measures regarding COVID-19 were found significantly more likely to being females (17.32 ± 2.78, t = -11.12, *p*<0.001); being young adult (20–29) (17.15 ± 2.62, *f* = 70.66, *p*<0.001); having higher education (17.25 ± 2.53, *f* = 266.15, *p*<0.001); being student (17.14 ± 2.64, *f* = 18.77, *p*<0.001); living in district town (17.32 ± 2.65, *f* = 125.61, *p*<0.001); being unmarried (17.13 ± 2.65, *f* = 91.18, *p*<0.001); being non-smoker (17.07 ± 2.79, t = -10.12, *p*<0.001); and being non-alcoholic (17.00 ± 2.85, t = -10.35, *p*<0.001) (**Table 1**).

### Risk factors of preventive COVID-19 behaviors

Several stepwise linear regression analyses were performed to analyze the factors associated with preventive COVID-19 behaviors. The analysis found participants with high number of comorbidities, being male, either divorced or widowed, consumed alcohol, smoked cigarettes, lived in villages and had no formal education reported lower preventive COVID-19 behaviors. Those participants with higher knowledge scores reported higher preventive COVID-19 behaviors (β = 0.053, *p*<0.001). However, the model predicted only 13.2 percent of the variation in performance of preventive COVID-19 behaviors (adjusted R^2^ = 0.132), and the overall model was significant (F = 59.0, *p*<0.001) (**Table 2**).

**Table 2:** Linear regression analysis of the variables associated with COVID-19 preventive behaviors.

Predictors	Unstandardized B	SE	Standardized B	95% CI	
				Lower	Upper
**Constant**	13.33	0.38		12.58	14.08
**Number of comorbidities**	-0.53	0.07	-0.08	-.66	-0.39
**Age**	-0.01	0.005	-.03	-.02	0.001
**Gender (female)**	.50	0.06	.09	.38	.62
**Knowledge**	.06	.01	.06	.04	.07
**Educational status**					
*No formal education*	-4.60	.23	-.22	-5.04	-4.16
*Primary school*	-2.26	.24	-.10	-2.72	-1.79
*Secondary school*	-.98	.14	-.07	-1.26	-.70
*Higher secondary level*	-.59	.09	-.06	-.76	-.42
*Tertiary education*	-				
**Occupational status**					
*Unemployed*	.04	.15	.002	-.26	.33
*Employed*	.21	.09	.03	.04	.38
*Retired*	.43	.33	.01	-.23	1.08
*Housewife*	.12	.11	.01	-.10	.34
*Student*	*-*				
**Residence**					
*Village*	-.85	.07	-.12	-1.00	-.70
*Sub-district town*	-.11	.09	-.01	-.28	.06
*District town*	.13	.07	.02	-.01	.27
*Divisional city*	*-*				
**Marital status**					
*Single*	*-*				
*Married*	-.10	.09	-.01	-.27	.08
*Divorced/widowed*	-.58	.25	-.02	-1.07	-.08
**Currently smoker (no)**	.31	.09	.04	.14	.48
**Alcohol use (no)**	1.14	.18	.06	.79	1.50

### District-wise distribution of COVID-19 knowledge and preventive behaviors

**Fig 1:**
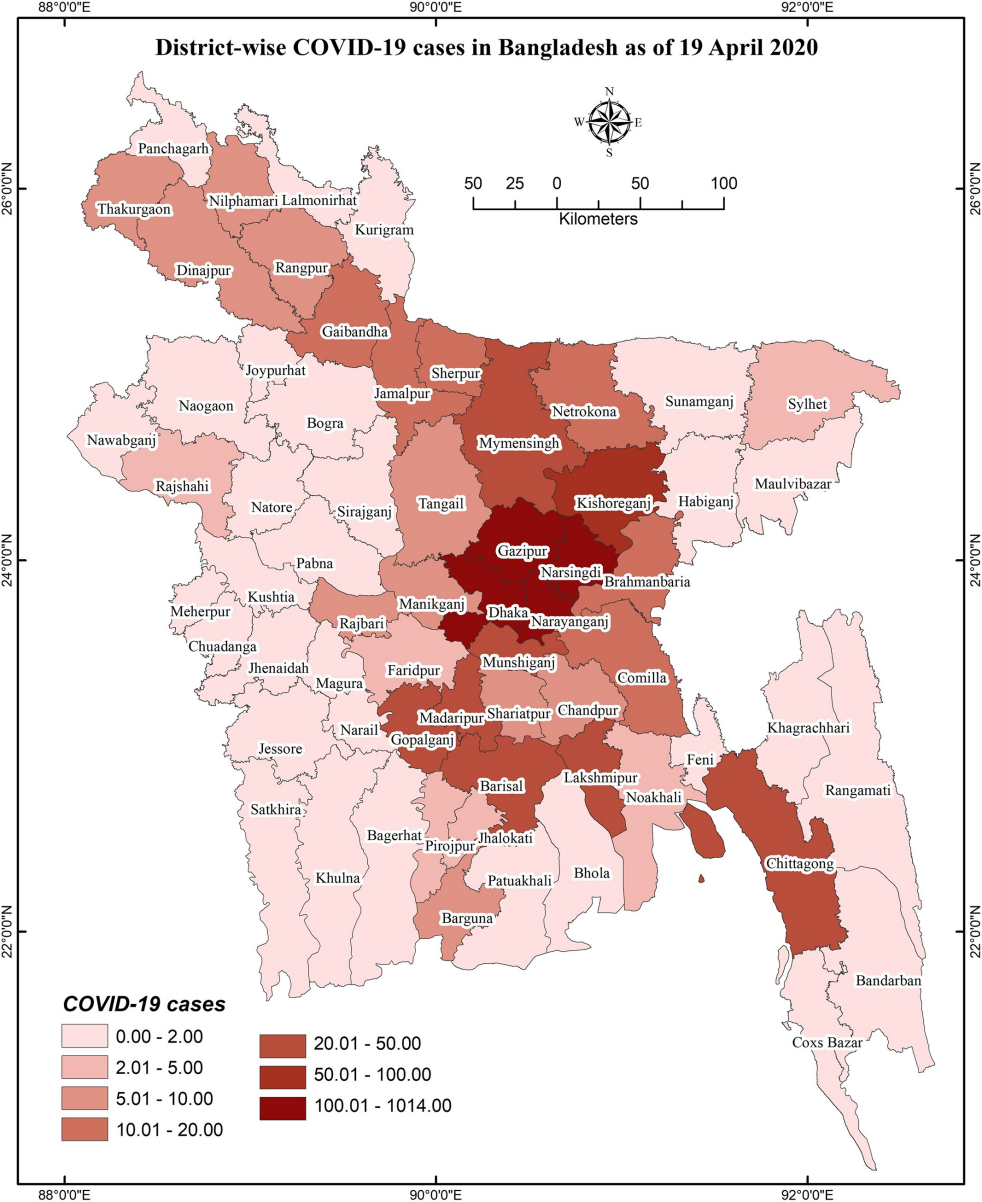
District-wise COVID-19 cases distribution in Bangladesh as of 19 April, 2020 [25].

**Fig 2:**
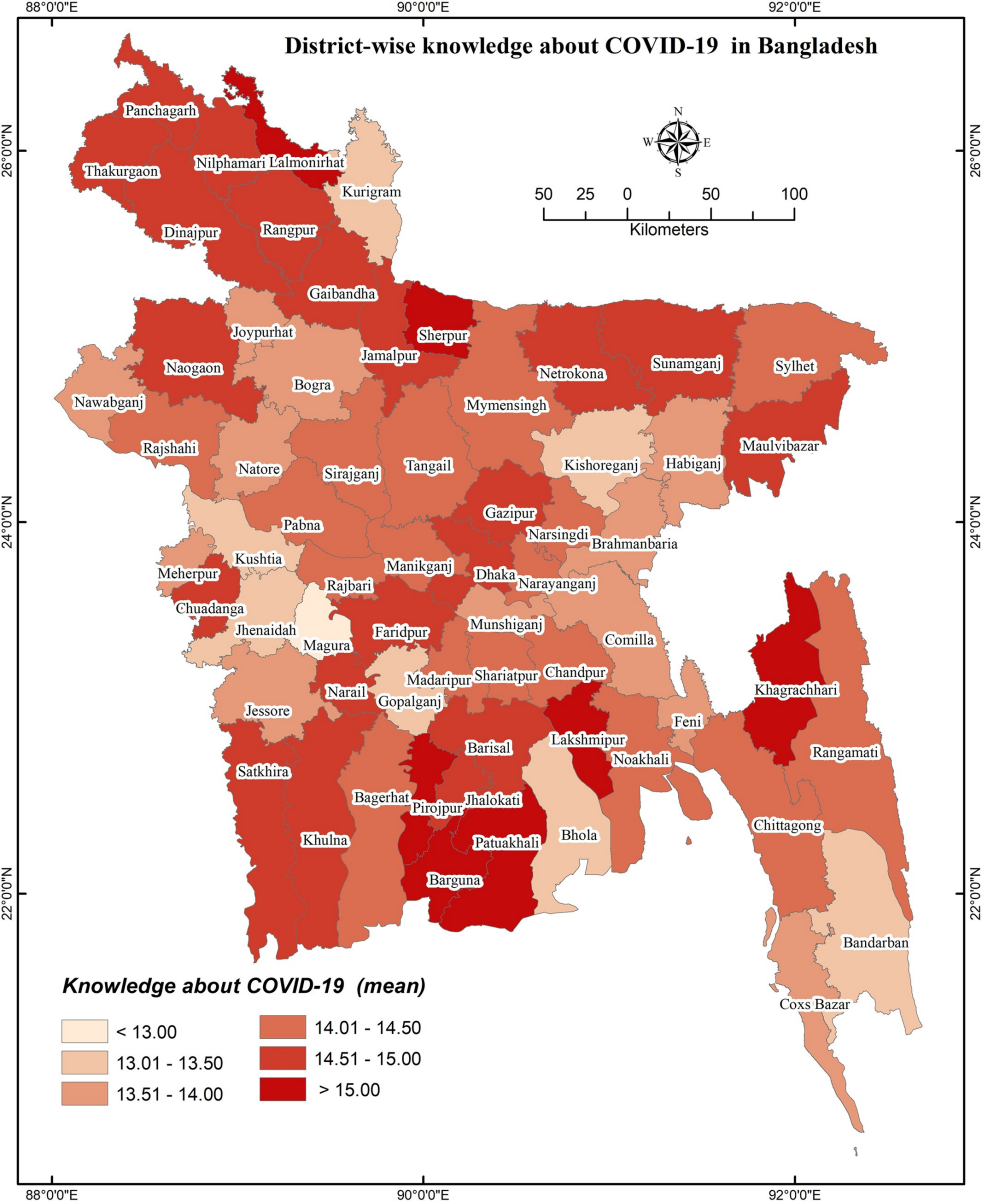
District-wise knowledge about COVID-19 in Bangladesh.

**Fig 3:**
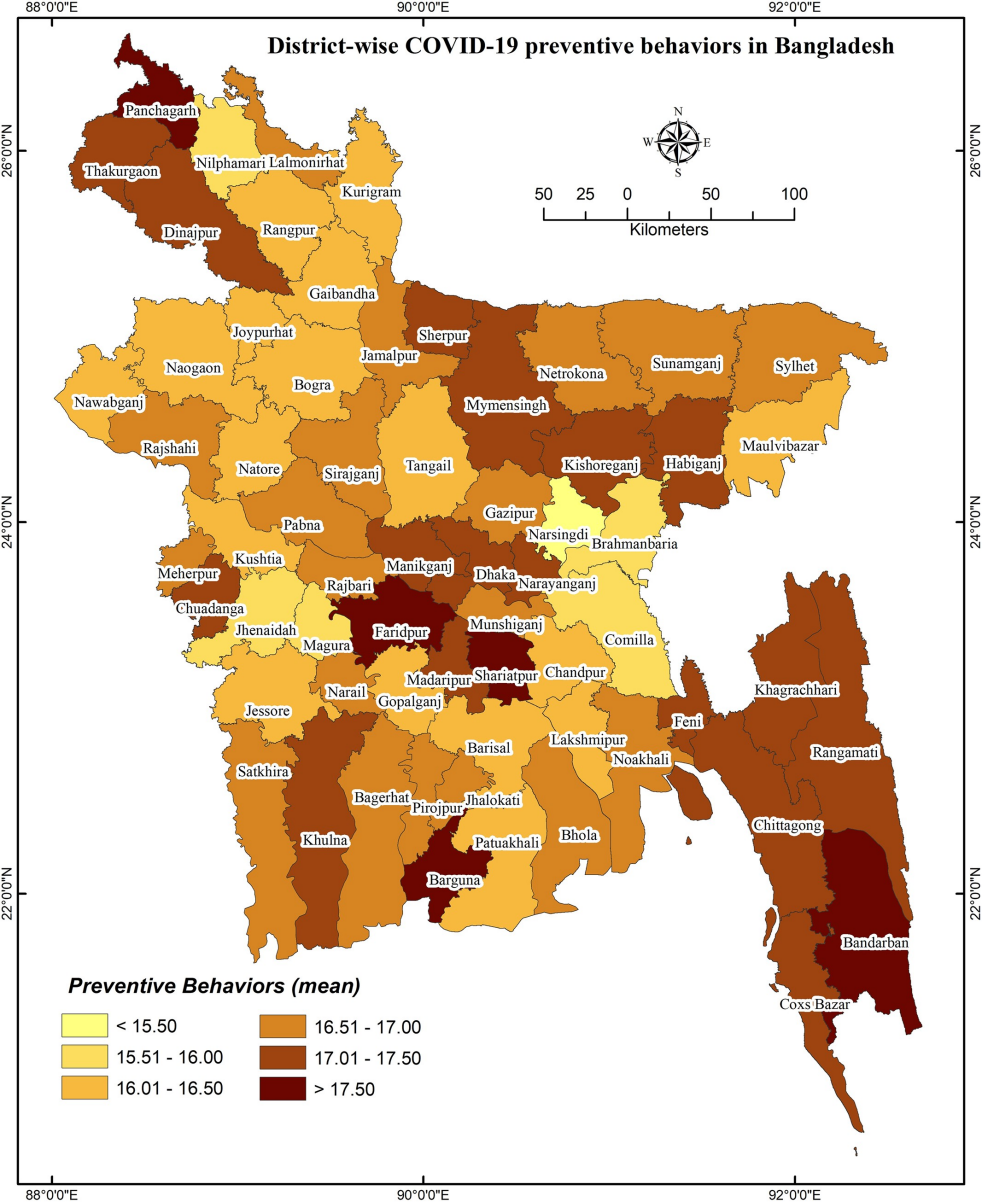
District-wise COVID-19 preventive behaviors in Bangladesh.

District-wise distributions of COVID-19 cases, knowledge, and preventive behaviors are presented in **Figs 1**–**3**, respectively. District-wide variations in spatial distributions of COVID-19 knowledge and preventive behaviors were statistically significant (i.e., χ^2^ = 32.995, *p*<0.001 and χ^2^ = 48.453, *p*<0.001, respectively). The higher level of COVID-19 knowledge was found in the districts of Lalmonirhat, Sherpur, Khagrachhari, Lakshmipur, Pirojpur, Jhalokhati, Patuakhali, Barguna, etc., whereas Magura, Kushtia, Gopalganj, Kishoreganj, Bhola, and Bandarban had lower knowledge (**Fig 2**). Besides, the districts Panchagarh, Barguna, Shariatpur, Faridpur etc. had been found with a higher level of COVID-19 preventive behaviors compared to Narsingdi, Magura, Jhenaidah, Cumilla and Brahmanbaria for which the level was lower (**Fig 3**).

**Fig 4:**
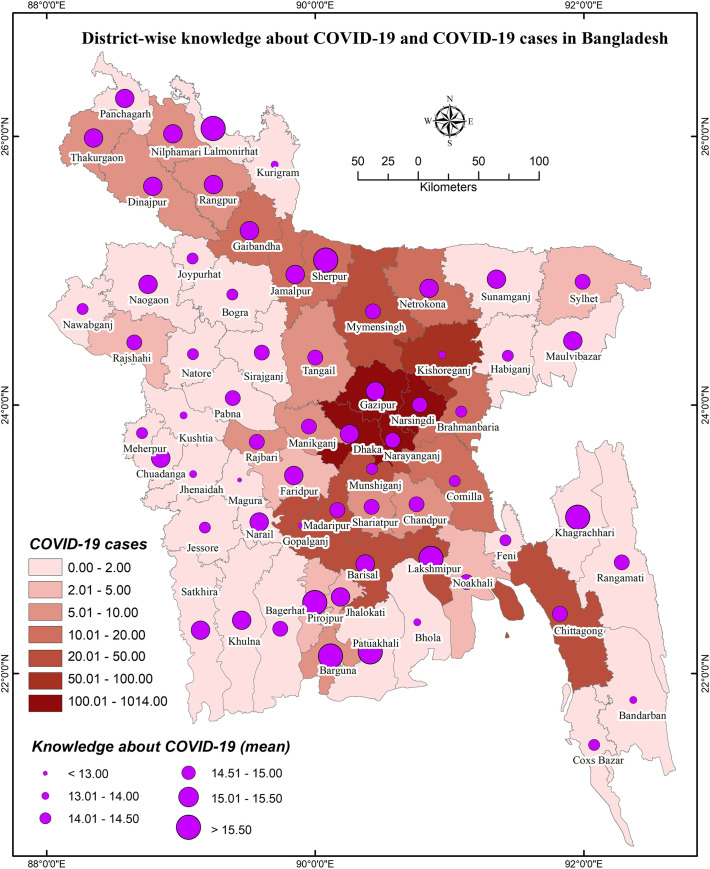
District-wise knowledge about COVID-19 and COVID-19 cases in Bangladesh.

**Fig 5:**
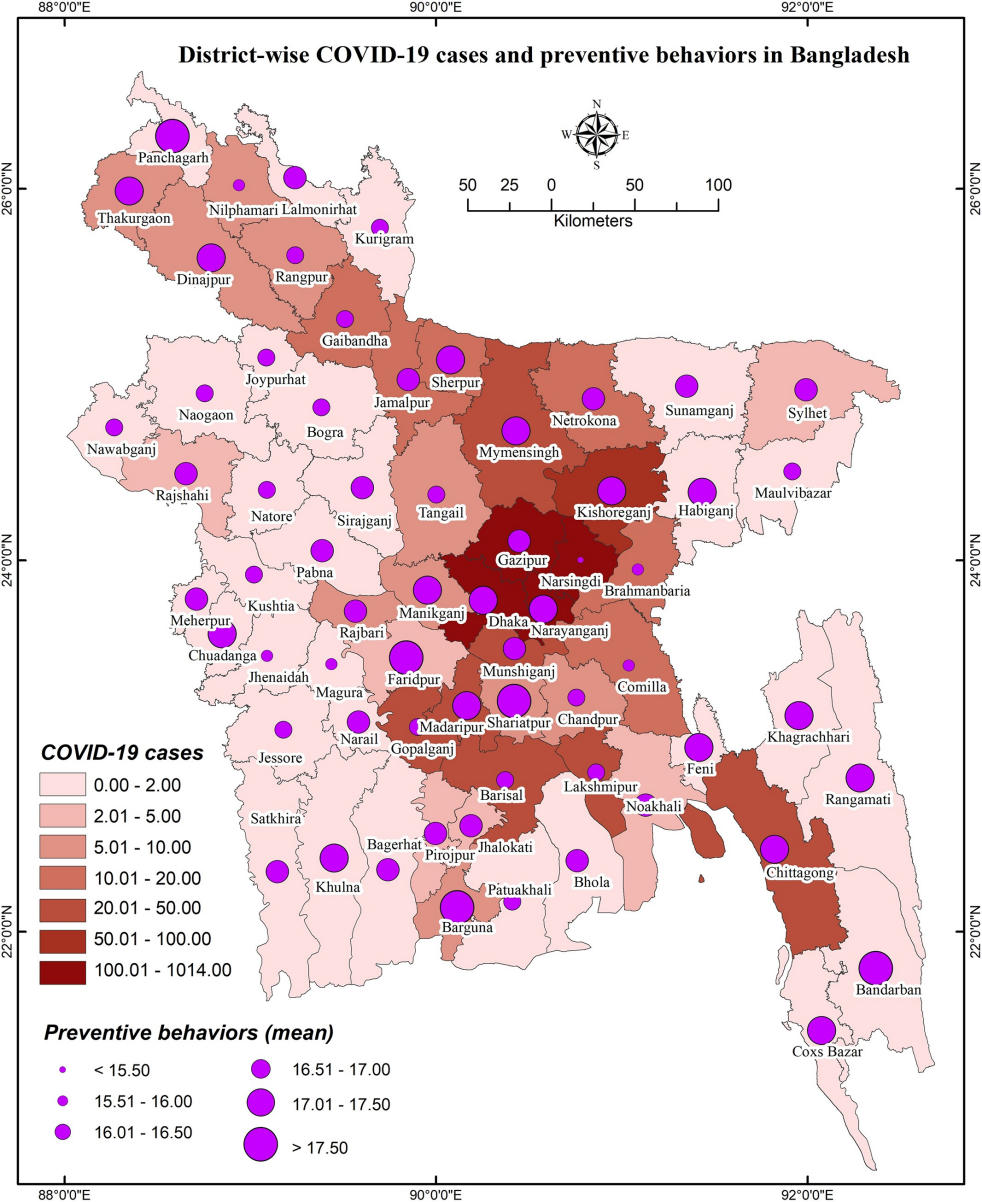
District-wise COVID-19 cases and preventive behaviors distribution in Bangladesh.

**Fig 6:**
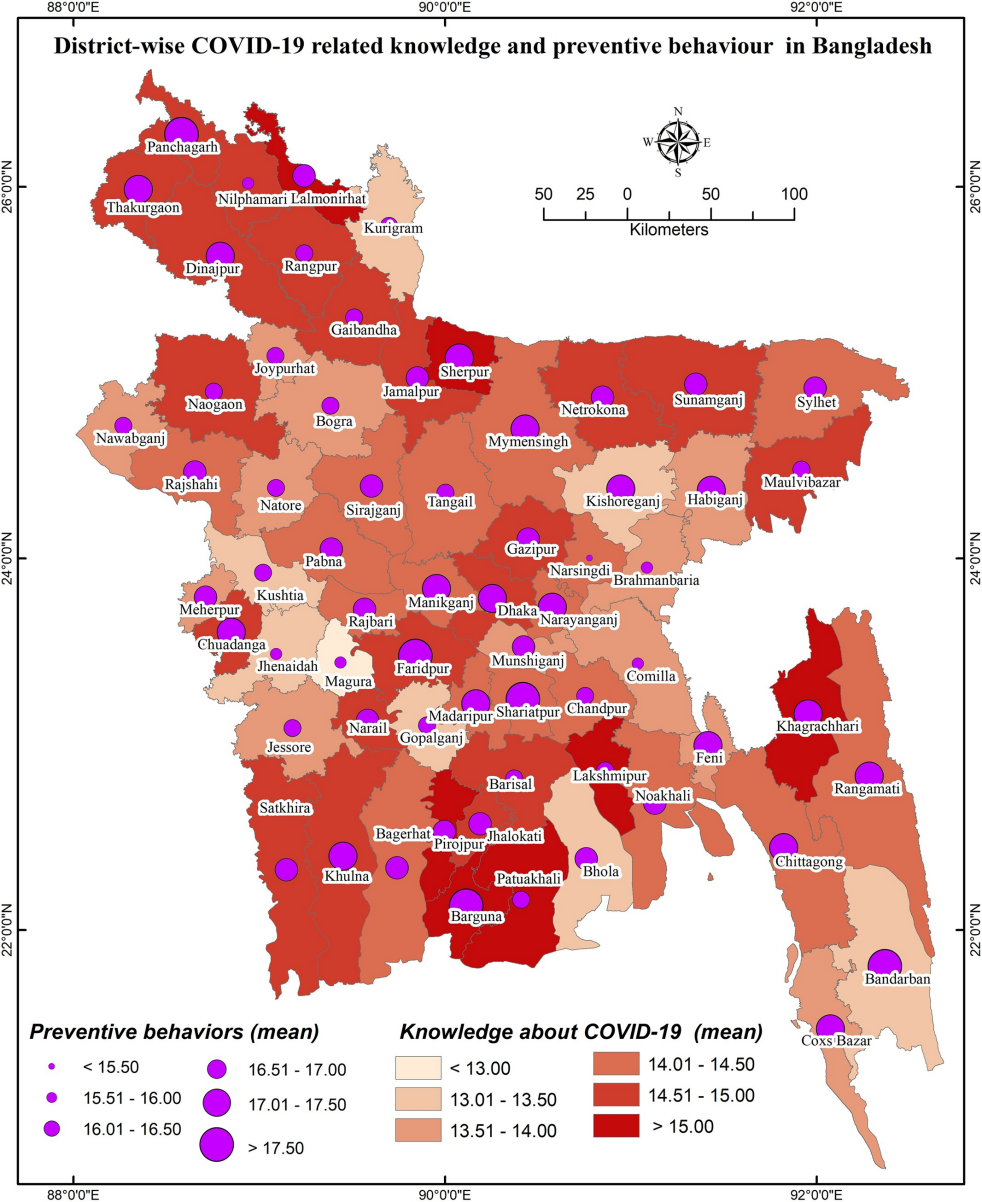
District-wise distribution of COVID-19 related knowledge and preventive behaviors in Bangladesh.

**Figs 4** and **5** represent the relationship of COVID-19 cases regarding knowledge about COVID-19 and COVID-19 related preventive behaviors, respectively; **Fig 6** illustrates the relationship between knowledge of COVID-19 and COVID-19 related preventive behaviors across the districts. Both knowledge of COVID-19 and COVID-19 preventive behaviors were heterogeneously distributed with the COVID-19 case number (i.e., no consistency in higher COVID-19 cases with higher knowledge or preventive behaviors were found) (**Fig 5**). Nevertheless, COVID-19 preventive behaviors were reported higher in those regions having higher knowledge about COVID-19 (**Fig 6**).

## Discussion

The current study represents one of the very few studies published assessing knowledge and preventive behaviors towards COVID-19 in the Bangladeshi context considering participants from diverse occupations [3, 9, 19–21]. The major strengths of the study are its large sample size and district-wise mapping, which provides a better insight of the scenario towards COVID-19 in Bangladesh.

Previous reports suggested that males’ irresponsible attitudes towards COVID-19 preventive measures accounted for them being more vulnerable to the disease than females [26, 27]. This study also found female participants with better knowledge and preventive behaviors than male ones. This findings were consistent with some of the previously conducted studies [2, 20], while in contrast with few studies where male participants had higher score on knowledge [28, 29]. It has been assumed that females are more conscious of preventive behaviors of specific issues like the ongoing COVID-19 pandemic [27]. In addition, knowledge and preventive behavior were found significantly higher among cigarette smokers compared to nonsmokers, but it was the opposite finding in case of alcohol users; this has not been previously reported in any other studies. Besides this, the respondents with high comorbidities had lower preventive behavior in this survey. The situation seems to be alarming because comorbidities are the most likely factors to aggravate the COVID-19 situation more severely [1]. Previous studies indicated that the level of knowledge was higher among the individuals living in urban areas [9, 28], which is similar to the present study. Easier accessibility of information in those areas than the suburbs can be one of the reasons behind this. Persons having higher degrees or education are more likely to have better knowledge and preventive behavior in this COVID-19 situation [2, 19], which is consistent with the present findings. But the present study also suggested having more knowledge about COVID-19 intensified the more preventive COVID-19 behaviors.

Regarding GIS-based distributions of COVID-19 knowledge and preventive behaviors, significant associations were found with district-wide spatial distributions. Districts of/near to the Rangpur division, coastal areas, and mountain area districts had higher levels of knowledge about COVID-19. But, preventive behaviors were better performed in the districts neighboring to the capital (Dhaka), Chittagong hill tracts, some of the northwest regions’ districts, etc. In addition, people’s fear of COVID-19 increased if they reported residing in the COVID-19 heavily exposed areas [30]. The fear of COVID-19 also significantly mediated the associations between perceived health status and sleeping problems, psychological distress, and COVID-19 preventive behaviors, as reported in a recent study among older adults [31]. Similarly, another study reported more engagement in preventive behaviors while the people were in higher fear or threat of COVID-19 infection [32], concluding perceived COVID-19 threat as a motivational factor of COVID-19 prevention [30]. Although it is expected to have better preventive behaviors in the areas with more COVID-19 cases, the present study did not discern that more COVID-19 affected regions had either more knowledge of COVID-19 or elevated levels of COVID-19 preventive behaviors. Likely aforementioned, the present spatial distribution also found the positive role of knowledge in preventive COVID-19 behaviors—people in these areas reporting more knowledge about COVID-19 were seen to practice more preventive behaviors.

The present study was cross-sectional in nature, and did not consider other important behavioral predictors [17] and this limits the generalizability of the findings. Furthermore, the study was conducted via an online platform, which leads to lower levels of non-educated individuals participating. Hence, the findings’ generalizability can be limited for the whole population, which is more applicable to the college-educated millennials. Lastly, there was no sufficient data for examining scientifically the association between ‘individual involvement in strategies’ and ‘effectiveness of strategies for control and prevention of disease outbreak’, which can be treated as one of the limitations. However, a follow up study is required to observe COVID-19 related preventive behaviors during time varieties considering fear related to COVID-19 issues which will help the government take necessary steps to control COVID-19 outbreak.

## Conclusions

The present study provides GIS-based COVID-19 knowledge and preventive behaviors for the first time in Bangladesh. According to the present findings, it is suggested that the government of Bangladesh should provide necessary efforts to supply appropriate health schooling regarding knowledge and preventive behaviors of COVID-19 at a community level and urgently apply the action plans to control the outbreak of COVID-19 pandemic. More importantly, individuals’ involvement in parallel to the government tactics will be obligatory to control and prevent the outbreak as the government has already failed to manage some of the crucial preventive aspects towards COVID-19, biomedical waste management, for example [33].

## Supporting information

S1 File (DOCX)
