# Supplementary material for: Knowledge and preventive behaviors regarding COVID-19 in Bangladesh: A nationwide distribution
Source: PLoS One. 2021 May 3;16(5):e0251151. doi: 10.1371/journal.pone.0251151 (PMC8092662; doi:10.1371/journal.pone.0251151)
Supplement: S1 File — (DOCX) [file pone.0251151.s001.docx]

**Socio-demographics**

**Your age (Write in number, e.g. 20)**

…………………………..

**Gender**

1. Male
2. Female

**Educational qualification**

1. No formal education
2. Primary school (Up to class 5)
3. Secondary school (class 6 to 10)
4. Higher secondary (class 11 to 12)
5. Tertiary education

**Occupation**

1. Unemployed
2. Employed
3. Retired
4. Housewife
5. Student

**Current district of residence (e.g. Dhaka)**

…………………………..

**Which type of administrative region are you living in?**

1. Village
2. Sub-district town
3. District town
4. Divisional city

**Marital status**

1. Single
2. Married
3. Divorced/widowed

**Do you smoke?**

1. Yes
2. No

**Do you consume alcohol?**

1. Yes
2. No

**Are you suffering from any of the following health-related issues? (You can choose more than one answer) (If you do not have any, please proceed to the next question)**

1. Diabetics
2. High blood pressure
3. Asthma/ Respiratory problem
4. Heart disease
5. Kidney problem
6. Cancer
7. Any other not listed/others

**Knowledge related questions (KRQ)**

**KRQ_1: ‘Spread of Infection’**

**Which of the following statements you think ‘TRUE’ in terms of spreading COVID-19? (You can choose more than one answer)**

1. Can spread from the COVID-19 affected persons through coughs or exhales
2. Can spread from the COVID-19 affected persons by touching others
3. Can spread from wild animals to humans
4. Can spread from the feces of the COVID-19 affected persons
5. Do not spread from companion animals or pets such as cats and dogs to humans
6. Do not spread through the parcels coming from COVID-19 affected countries

**KRQ_2: ‘Symptomology’**

**Which of the following symptoms you think ‘TRUE’ in the case of COVID-19? (You can choose more than one answer)**

1. Incubation period for COVID-19 ranging from 2-14 days
2. Some people may not develop any symptoms
3. The most common symptoms of COVID-19 are fever, tiredness, and dry cough.
4. Respiratory problems/ Pneumonia will never develop
5. Some patients may have aches and pains, nasal congestion, runny nose, sore throat or diarrhea.
6. Those with underlying medical problems like high blood pressure, heart problems or diabetes, are less likely to develop serious illness (e.g organ failure)

**KRQ_3: ‘Prevention’**

**Which of the following preventive measures you think ‘TRUE’ that can be taken in the case of COVID-19? (You can choose more than one answer)**

1. Washing hands regularly for 20 seconds
2. Touching eyes, nose and mouth
3. Wearing masks
4. Avoiding close contact from the affected persons
5. Maintain at least 1-meter (3 feet) distance between yourself and anyone who is coughing or sneezing.
6. Maintain home quarantine if you feel unwell and isolate the affected person

**KRQ_4: ‘Treatment’**

**What are the treatments you think ‘TRUE’ that one can follow for COVID-19? (You can choose more than one answer)**

1. Taking pills like antibiotics when you have fever.
2. To date, there is no vaccine and no specific antiviral medicine to prevent or treat COVID-2019.

**Preventive Behavior related questions (BRQ)**

**BRQ_1: How often do you clean your hands with an alcohol-based hand rub or wash them with soap and water?**

1. Never
2. Seldom
3. Sometimes
4. Often
5. Almost always

**BRQ_2: How often do you Practice respiratory hygiene (covering your mouth and nose with your bent elbow or tissue when you cough or sneeze)?**

1. Never
2. Seldom
3. Sometimes
4. Often
5. Almost always

**BRQ_3: How often do you maintain at least 1-meter (3 feet) distance between yourself and anyone who is coughing or sneezing?**

1. Never
2. Seldom
3. Sometimes
4. Often
5. Almost always

**BRQ_4: How often do you stay home if you feel unwell?**

1. Never
2. Seldom
3. Sometimes
4. Often
5. Almost always
